# Author Correction: Low frequency transcranial electrical stimulation does not entrain sleep rhythms measured by human intracranial recordings

**DOI:** 10.1038/s41467-018-03392-9

**Published:** 2018-02-28

**Authors:** Belen Lafon, Simon Henin, Yu Huang, Daniel Friedman, Lucia Melloni, Thomas Thesen, Werner Doyle, György Buzsáki, Orrin Devinsky, Lucas C. Parra, Anli Liu

**Affiliations:** 1 Department of Biomedical Engineering, City College of New York, 160 Convent Ave, New York, NY 10031 USA; 2 New York University Comprehensive Epilepsy Center, 223 East 34th Street, New York, NY 10016 USA; 3 Department of Neurology, New York University School of Medicine, 240 East 38th St, 20th Floor, New York, NY 10016 USA; 4 Department of Neuroscience, Max Planck Institute for Empirical Aesthetics, Gruneburgweg 14, 60322 Frankfurt am Main, Germany; 5 Department of Physiology and Neuroscience, St. George’s University, St. George’s, Grenada; 6 Department of Neurosurgery NYU School of Medicine, 530 1st Avenue, Suite 7W, New York, NY 10016 USA; 7 New York University Neuroscience Institute, 450 East 29th St, New York, NY 10016 USA

Correction to: *Nature Communications* 10.1038/s41467-017-01045-x, published online 31 October 2017

It has come to our attention that we did not specify whether the stimulation magnitudes we report in this Article are peak amplitudes or peak-to-peak. All references to intensity given in mA in the manuscript refer to peak-to-peak amplitudes, except in Fig. 2, where the model is calibrated to 1 mA peak amplitude, as stated. In the original version of the paper we incorrectly calibrated the computational models to 1 mA peak-to-peak, rather than 1 mA peak amplitude. This means that we divided by a value twice as large as we should have. The correct estimated fields are therefore twice as large as shown in the original Fig. 2 and Supplementary Fig. 11. The corrected figures are now properly calibrated to 1mA peak amplitude. Furthermore, the sentence in the first paragraph of the Results section ‘Intensity ranged from 0.5 to 2.5 mA (current density 0.125–0.625 mA mA/cm^2^), which is stronger than in previous reports’, should have read ‘Intensity ranged from 0.5 to 2.5 mA peak to peak (peak current density 0.0625–0.3125 mA/cm^2^), which is stronger than in previous reports.’ These errors do not affect any of the Article’s conclusions. Correct versions of Fig. 2 and Supplementary Fig. 11 are presented below as Figs. 1, 2.

 **Fig. 1**

 **Fig. 2**

