# Supplementary material for: Low frequency transcranial electrical stimulation does not entrain sleep rhythms measured by human intracranial recordings
Source: Nat Commun. 2017 Oct 31;8:1199. doi: 10.1038/s41467-017-01045-x (PMC5662600; doi:10.1038/s41467-017-01045-x)
Supplement: Supplementary file 1 — Supplementary Information [file 41467_2017_1045_MOESM1_ESM.pdf]

SUPPLEMENTARY FIGURES

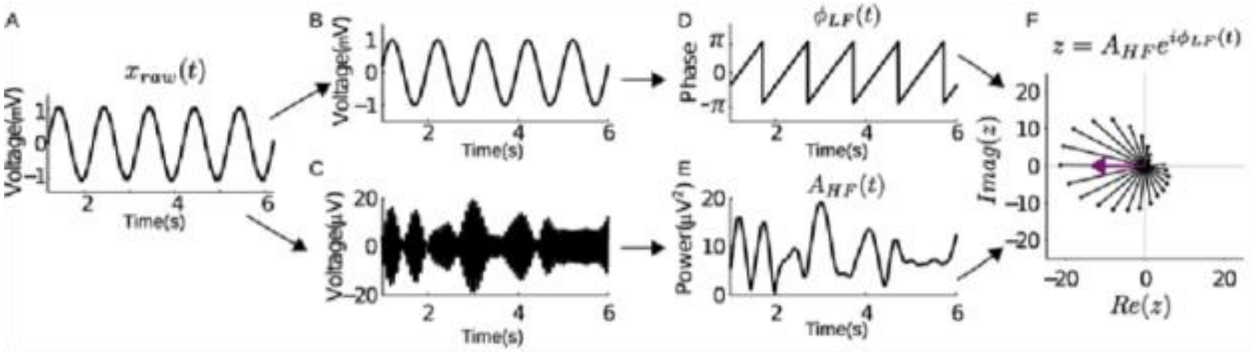

Supplementary Figure 1: Steps in the computation of the modulation index for the coupling between LF-stimulation and spindle rhythm. The raw signal (A) is manipulated to obtain the low frequency phase ($\varphi_{LF}(t)$) and the high frequency ($A_{HF}(t)$) amplitude. The low frequency stimulation signal is obtained by harmonic subtraction, where a sinusoid of the same frequency as the 1Hz-stimulation is fitted to the raw signal and subtracted from it (B). The stimulation phase (D) is calculated by taking the angle of the Hilbert transform of (B). The spindle activity (C) is obtained by filtering the raw data (A) with a bandpass Morlet wavelet filter centered at 14Hz. The filtered signal is complex and the absolute value of the signal represents the instantaneous magnitude at 14 Hz (E). The complex time series constructed as, $z(t) = A_{HF}(t)\ e^{i\ \phi LF(t)}$ is shown in (F), where the length of each point represents the amplitude of the signal (E) in the first second and the phase is given by (D). The modulation index is plotted in magenta.

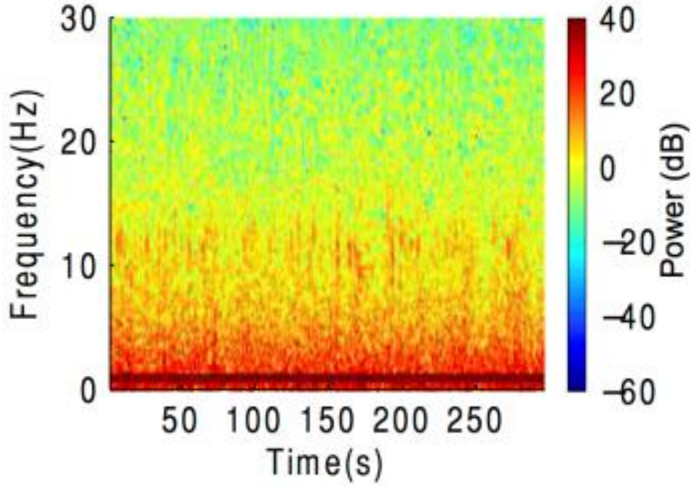

Supplementary Figure 2. Spectrogram of the raw data during TACS for subject S9 during sleep, for electrode G33. Stimulation was applied at 1 Hz, which is clearly visible here as a line artifact in the 1Hz band. Endogenous spindle rhythm is present at 14 Hz.

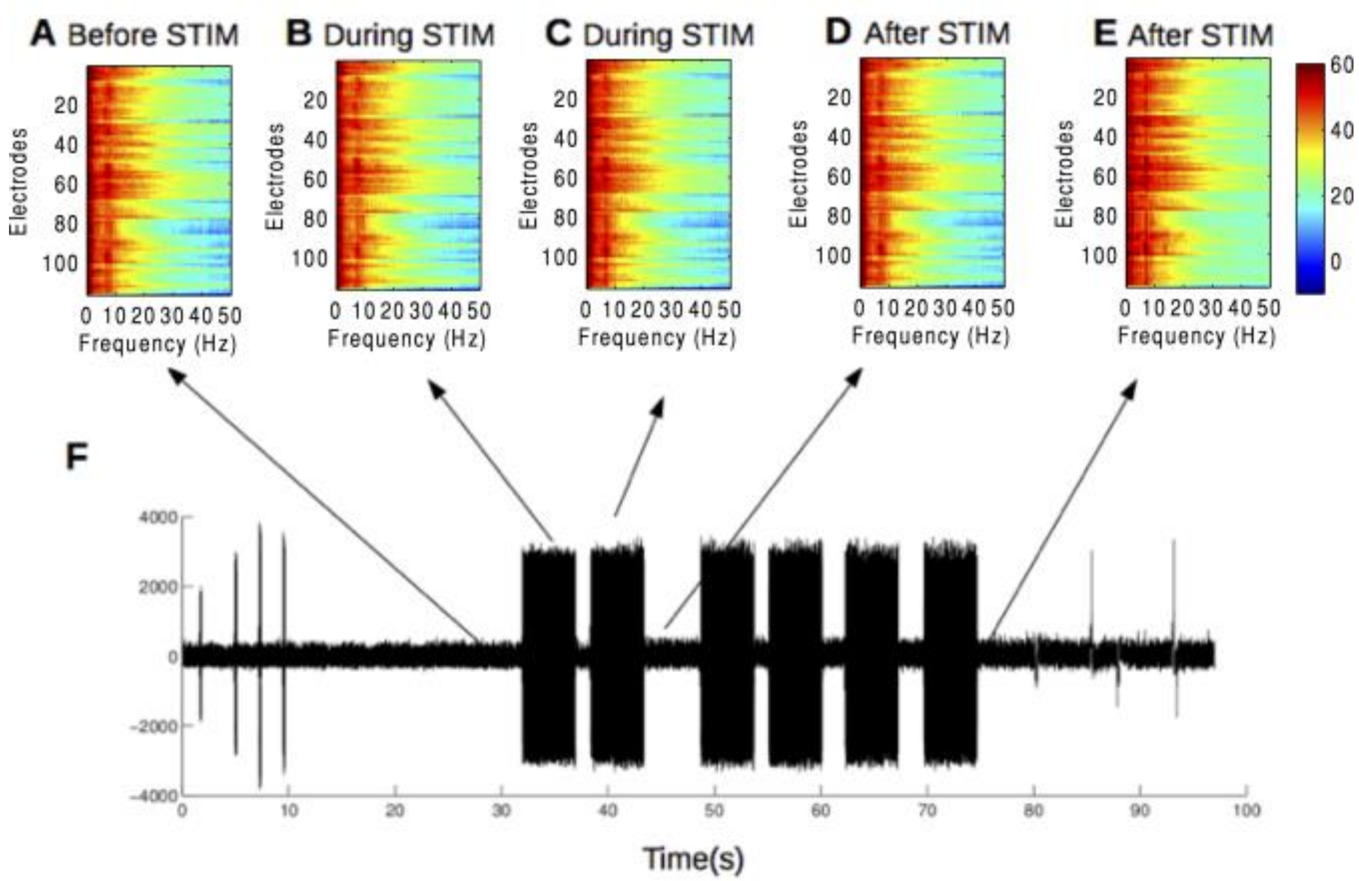

Supplementary Figure 3. Raw spectrum across iEEG electrodes before, during, and after stimulation blocks during NREM sleep. Raw spectrogram for one subject (S9). Stimulation was applied in 5-minute blocks, and acute effects of TACS were assessed. The raw spectrum in this exemplary electrode demonstrates that power of slow wave, theta, and spindle band oscillations during stimulation were comparable to activity before and after stimulation, suggesting that there were no acute changes in overall power (color bar units: dB). The lower trace is a raw voltage trace of the stimulation (units in y-axis: mV).

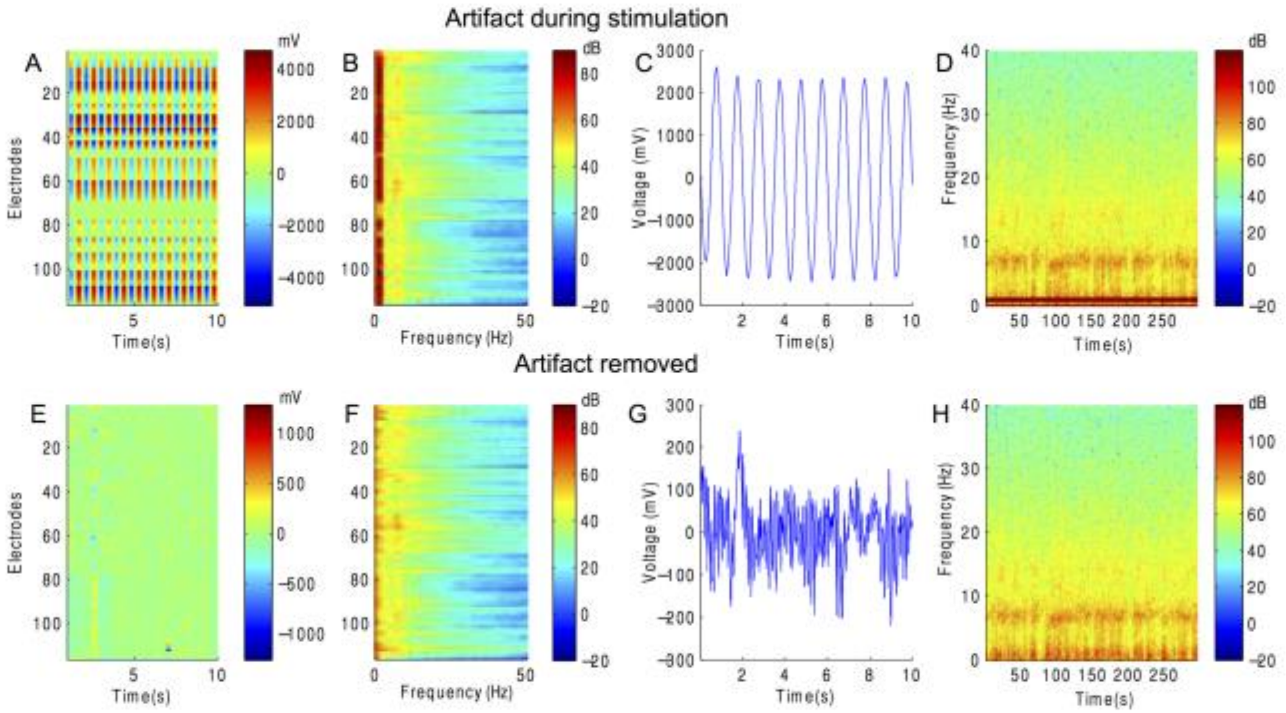

Supplementary Figure 4: Artifact analysis and removal. Representative analysis for subject S8. Upper row shows frequency and time domain characteristics of the TACS artifact. The lower row shows the same data after removing the artifact. (A/E) Voltage during 10 seconds of stimulation for all electrodes in S8 with color indicating voltage in units of mV. (B) Power for frequencies 0-50Hz for all electrodes with color indicating dB. (C/G) Trace of an exemplary electrode. Clearly the sinusoidal stimulation dominates and the iEEG signal is not visible prior to subtraction, but there is no evident artifact left after artifact removal. (D/H) The spectrogram for the same electrode show a large stimulation artefact as 1Hz, but no evident harmonics. Nevertheless, harmonics are fit up to 40Hz to assure that there is no phase-locked activity in the bands of interest. The spectra from the top and bottom rows look nearly identical (except that the dominant 1 Hz TACS artifact that has been removed).

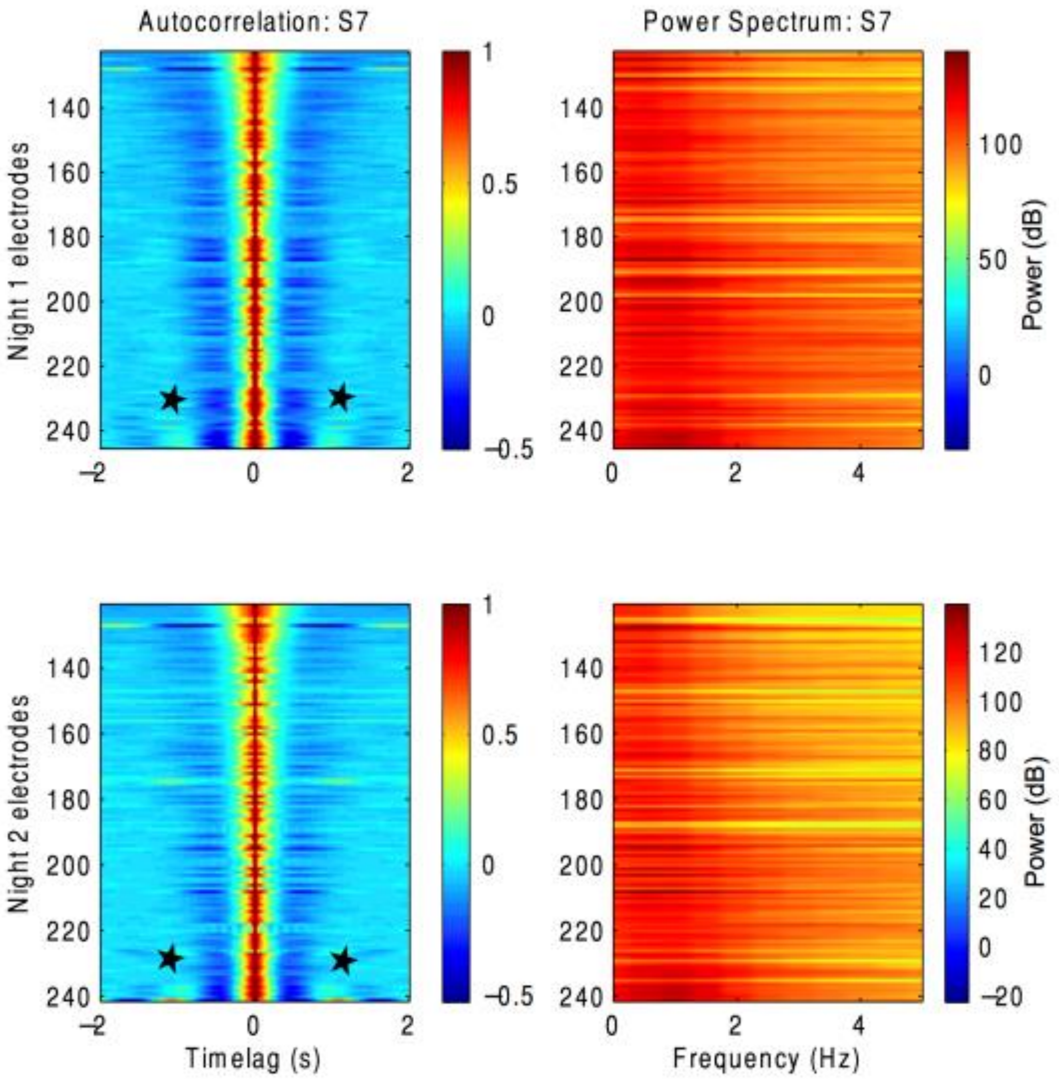

Supplementary Figure 5. Autocorrelation and power spectrum for all electrodes on subject S7 showing very low temporal coherence of slow wave activity with correspondingly broad spectrum. Electrodes are sorted strength of 1Hz peak (relative to activity <1Hz). Left row shows the autocorrelation during two different nights (autocorrelation coefficient has no units). Only a few electrodes show a second peak in the autocorrelation at time lag +/- 1s indicating a rhythmicity of the slow wave activity (electrodes 235-240 at the bottom of each panel with the highest electrode number have a symmetric peak at +/-1s marked with black stars). For those electrodes, a 1Hz peak is discernible in the power spectrum. For most electrodes, however, coherence is less than 1s indicating that slow wave activity are unitary events. See Supplementary Note 1.

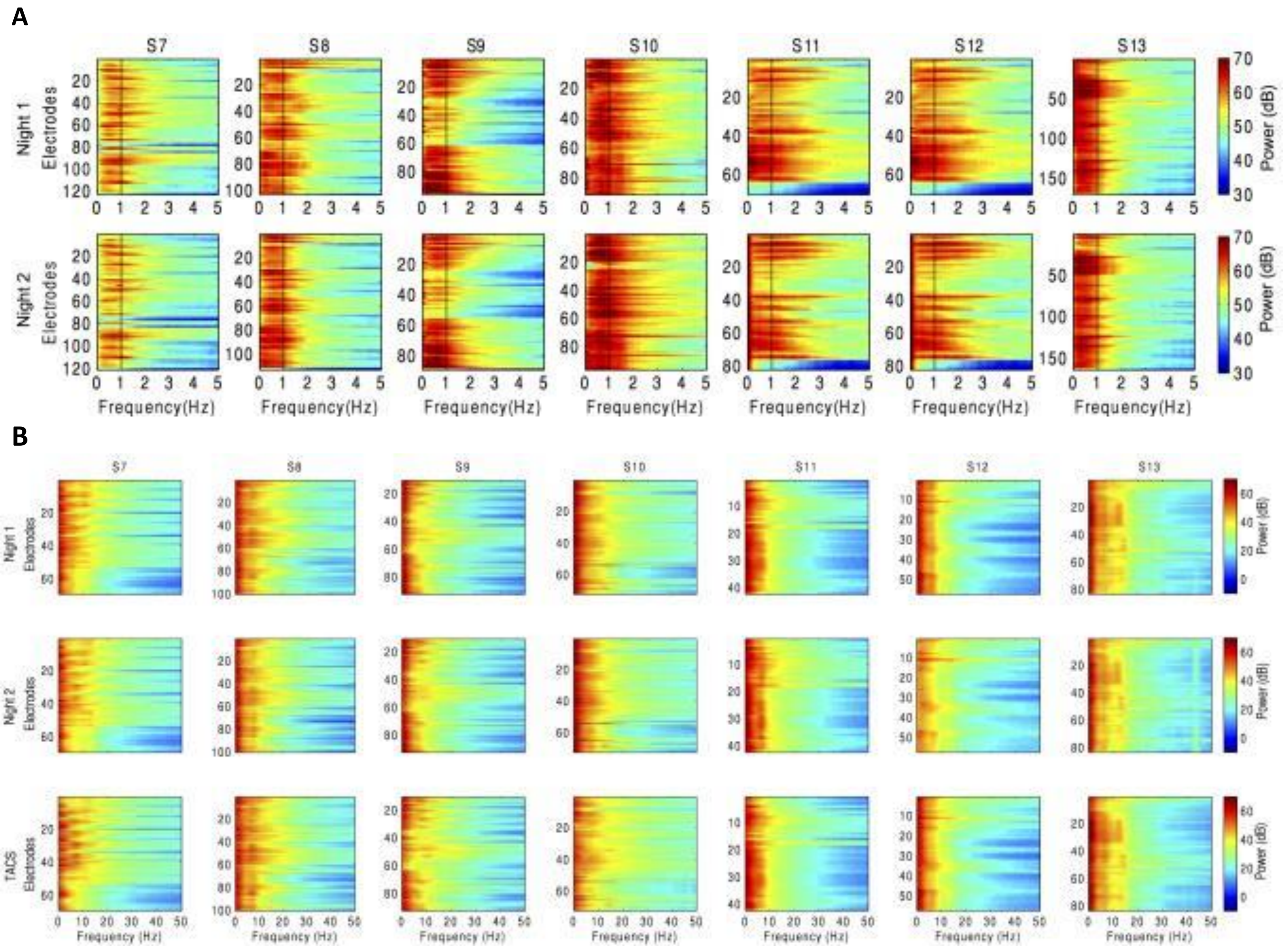

Supplementary Figure 6. Raw spectrogram of NREM sleep segments collapsed across electrodes. (A) These spectrograms demonstrate that slow-wave activity is broad-band without a well-defined spectral peak. 1Hz is indicated with a line as a reference. (B) These spectrograms demonstrate that NREM sleep during endogenous sleep and TACS are similar in quality, and that stimulation artifact, including harmonics up to 40Hz have been successfully removed. See Supplementary Note 1.

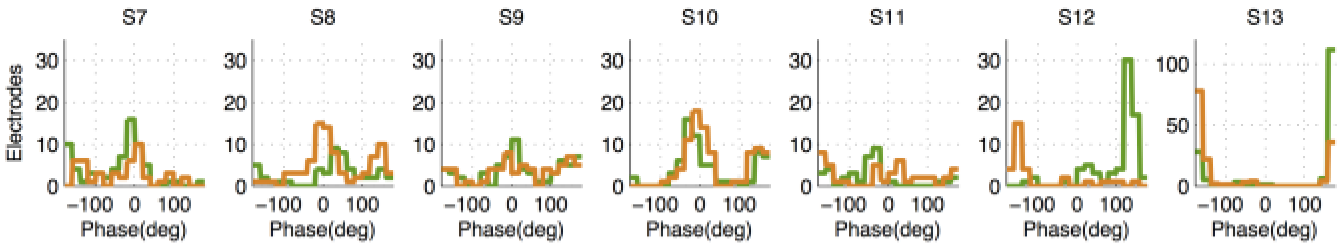

Supplementary Figure 7. Preferred phase of fast spindle (14Hz) activity amplitude relative to endogenous slow oscillation analyzed here with 0.75 Hz as center frequency. Histogram of the preferred phase for all electrodes with significant PAC for two different nights (Night 1, green; Night 2, orange). Each histogram corresponds to a different subject. See Supplementary Note 2.

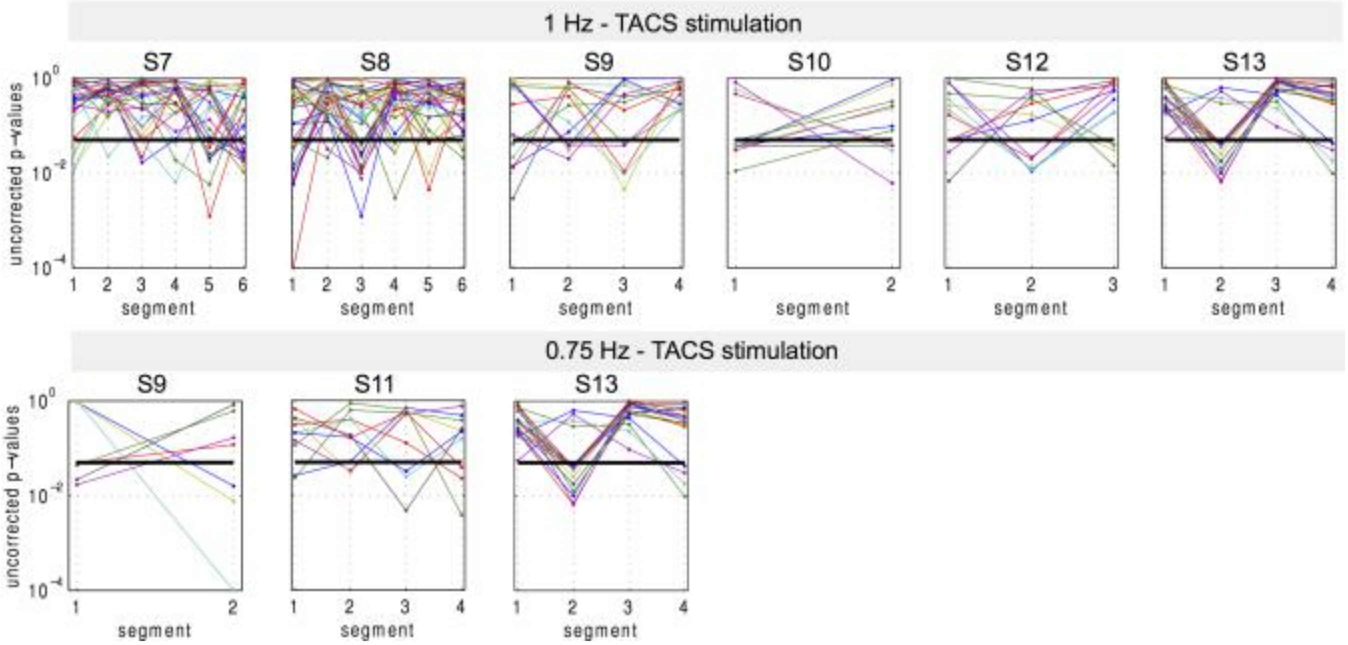

Supplementary Figure 8. P-values of PAC (14Hz amplitude to TACS phase) before multiple comparisons during TACS stimulation. The uncorrected p-values are shown in a logarithmic scale. Only electrodes that have an uncorrected p-value<0.05 during at least one stimulation segment are displayed. Each electrode is shown in different color to better identify it across segments. Black line indicates uncorrected p-value of 0.05. Note that electrodes which reach significance during one segment of stimulation do not remain significant across subsequent segments of stimulation. Only two electrodes survive multiple comparisons (subject S8, segment 1 of 1Hz TACS; S9, segment 2 of 0.75 Hz TACS). None of the electrodes that reach significance (before multiple comparisons correction) present stable entrainment across stimulation segments.

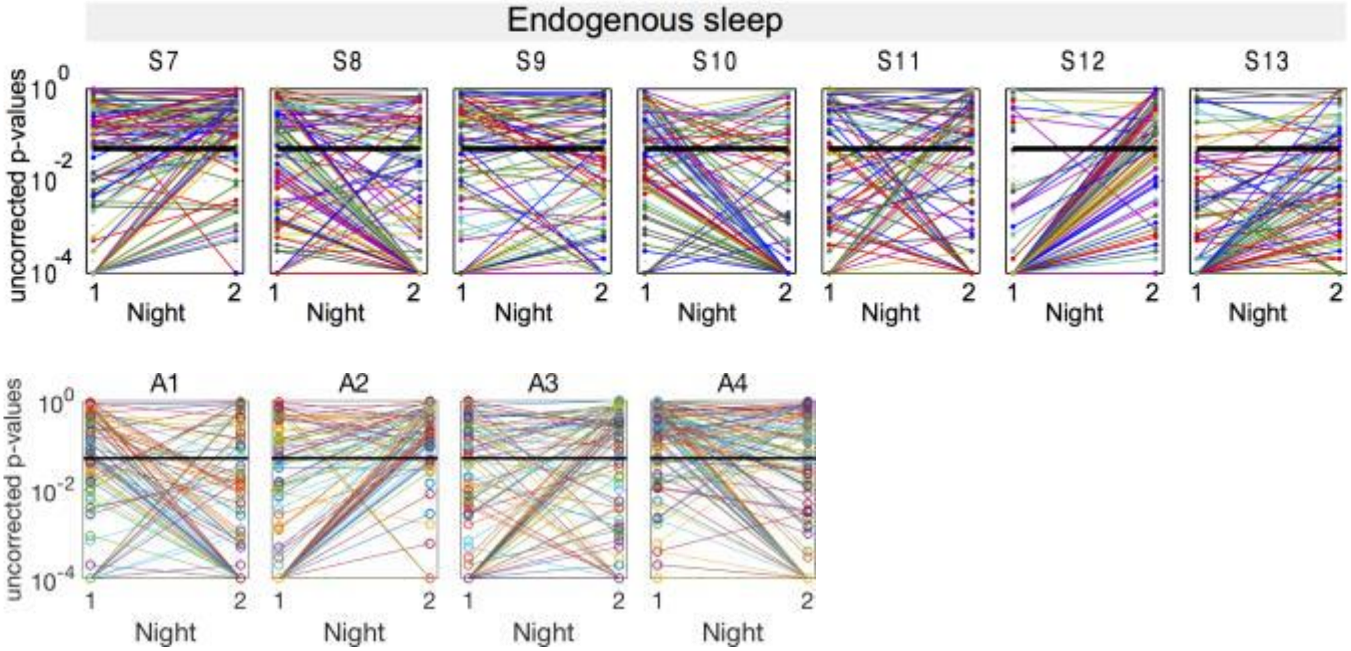

Supplementary Figure 9. P-values of PAC (14 Hz amplitude to endogenous SO phase) before multiple comparisons during endogenous sleep. The uncorrected p-values are shown in a logarithmic scale. Only electrodes that have uncorrected p-values<0.05 during one of both nights (at least) are displayed. Each electrode is shown in different color to better identify it across segments. Black line indicate p-value of 0.05. During night 1, 504 electrodes are significant after FDR correction and 480 electrodes for night 2 (Supplementary Table 2). Note that many of the electrodes which demonstrate spindle entrainment during night 1 also demonstrate night 2.

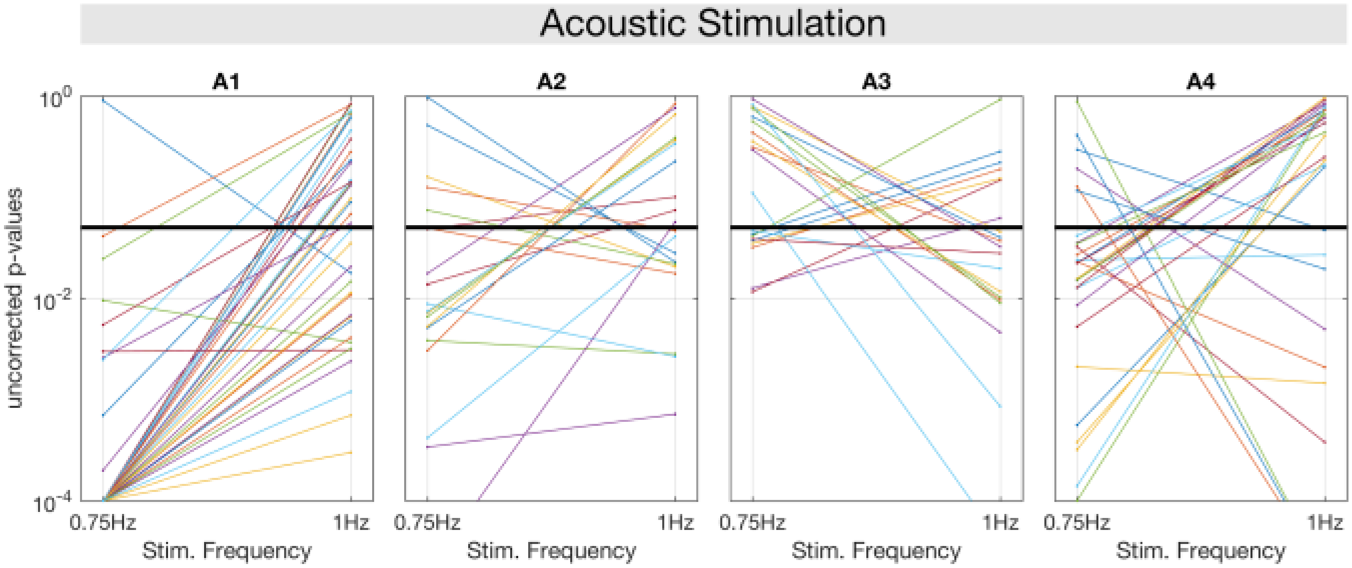

Supplementary Figure 10: P-values of PAC (14 Hz amplitude to induced SO phase) before multiple comparisons during acoustic stimulation. The uncorrected p-values are shown in a logarithmic scale. Only electrodes that have uncorrected p-values<0.05 during one of both stimulation segments (at least) are displayed. Each electrode is shown in different color to better identify it across segments. Black lines indicate p-value of 0.05.

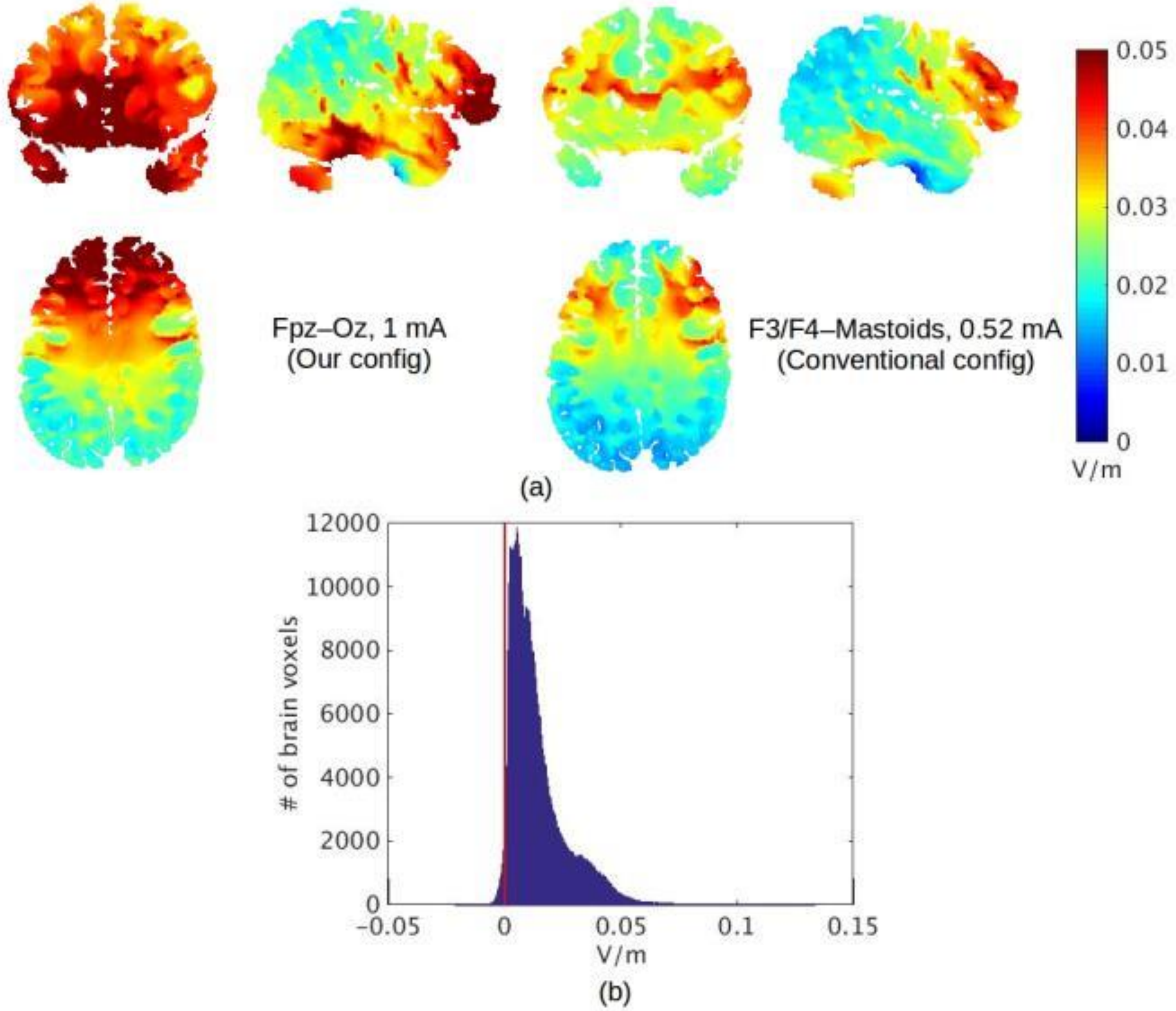

Supplementary Figure 11. Comparison of electric field intensities for our protocol to the conventional stimulation protocol. (a) Electric field distribution generated by calibrated computational models for our stimulation protocol (Fpz-Oz, 1 mA) and conventional protocol (F3/F4-Mastoids, 0.52 mA). (b) Histogram of the difference of the electric field between the two protocols measured across the brain as in (a). Values (blue to the right of the red vertical line indicates that our stimulation protocol was stronger, which is the case of 98% of locations in the brain, including in the frontal and midline depth locations.

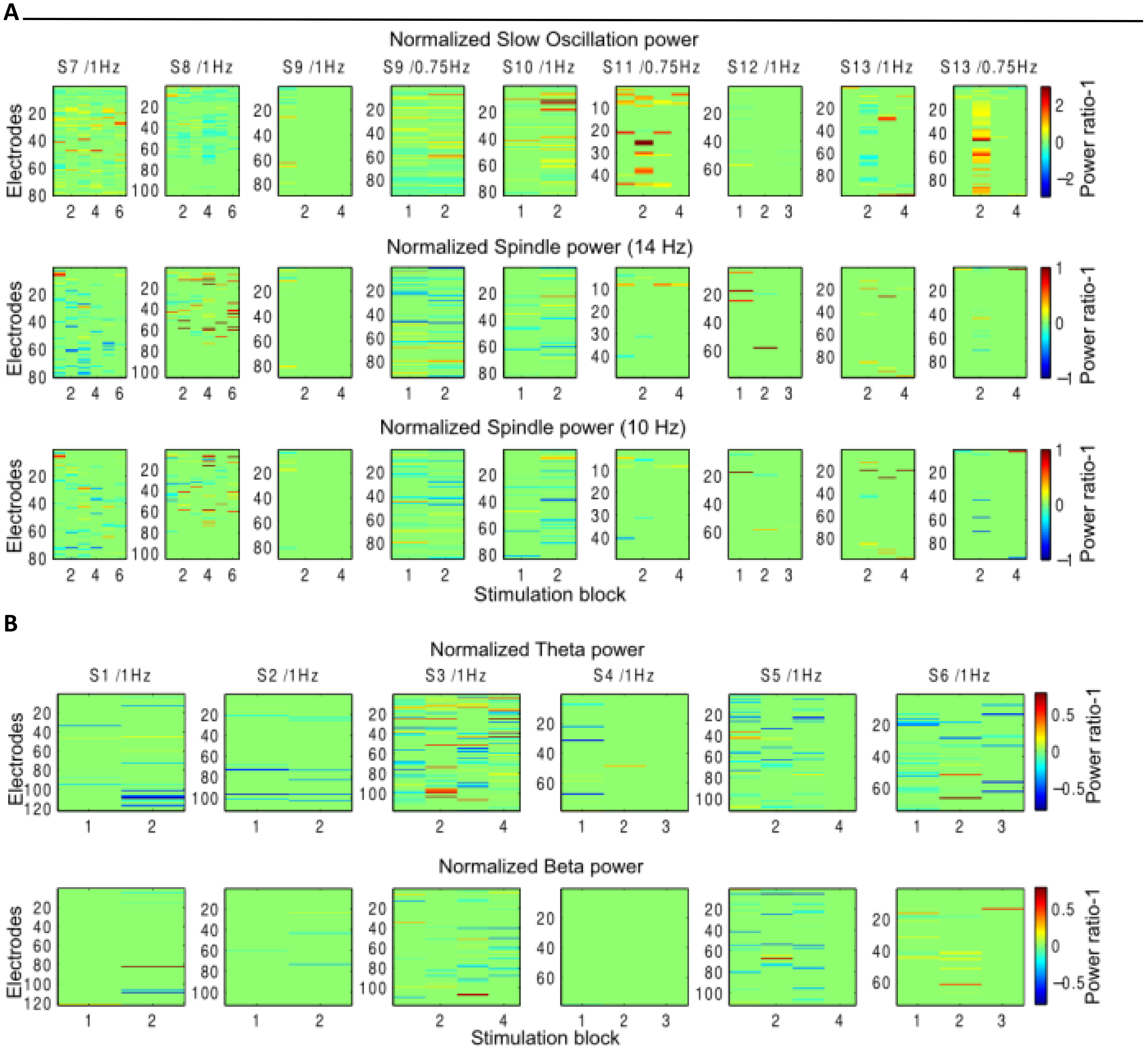

Supplementary Figure 12. Power changes after each TACS block normalized power before simulation. Power is measured for each electrode in 30 seconds following stimulation and normalized to the beginning of the recording session (30 seconds preceding the first block of stimulation). **(A)** TACS during NREM sleep. Upper row shows the power in the slow oscillation band (0.5 Hz-1 Hz) for all electrodes. Lower row displays the power for all electrodes in the fast and slow spindle bands. **(B)** TACS during wakeful rest. Upper row shows the power in the theta band (4 Hz-7 Hz) averaged during 30 seconds for all electrodes. Lower row displays the power for all electrodes in the beta frequency band (15 Hz- 25 Hz) averaged over time. Color indicates fraction increase (red) or decrease (blue) over baseline with numerical value indicating ratio-1. Electrodes shown in green have no statistical significant difference with baseline. The main observation is that changes, while significant, are inconsistent in sign within and across subjects.

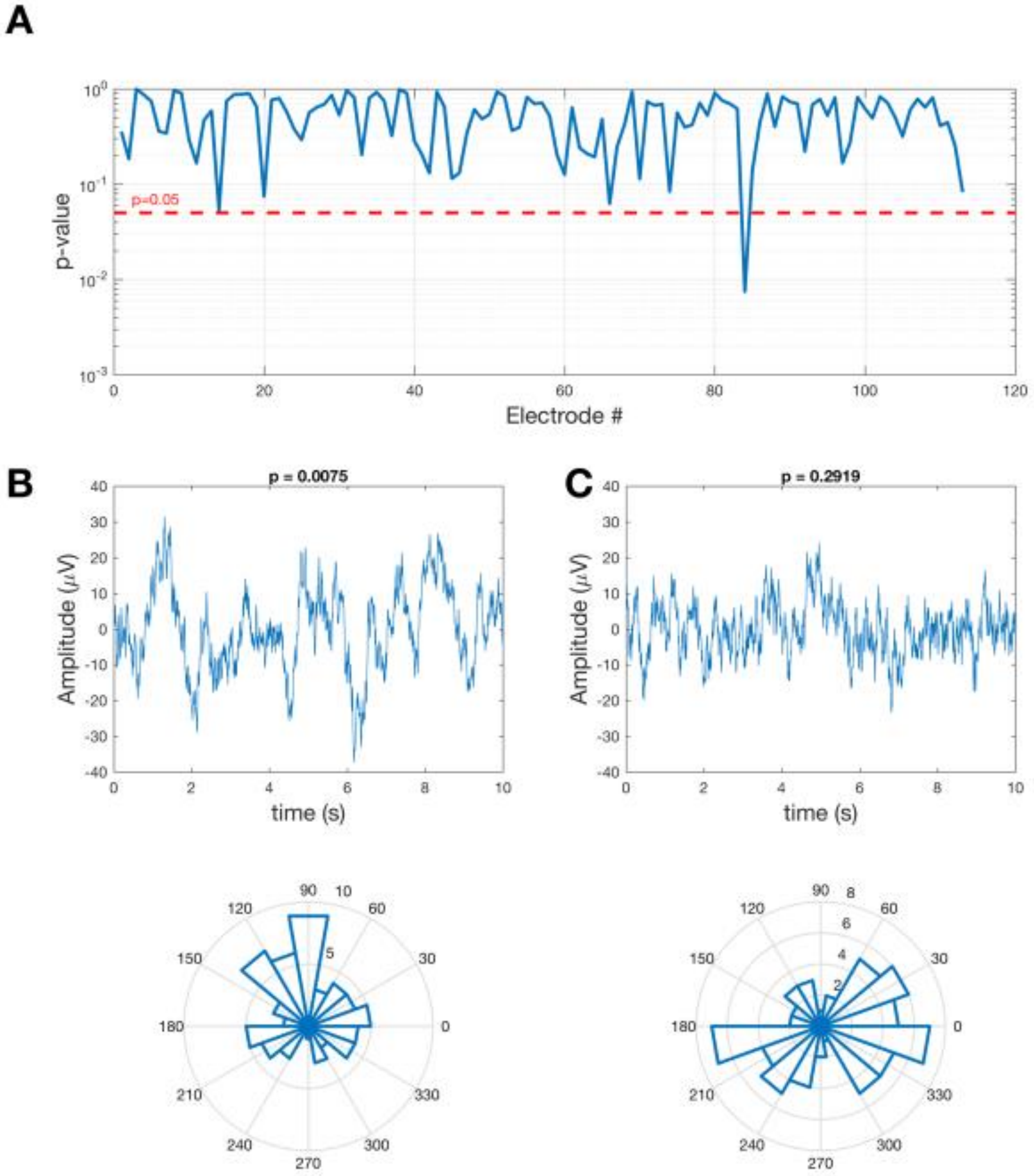

Supplementary Figure 13. Inter-trial phase coherence immediately after 0.75Hz trapezoidal stimulation. A) Uncorrected p-values for computed phase coherence (Rayleigh test for non-uniformity. B-C) Example averaged trace (top) and angle histograms (bottom) for computed sinusoidal fits (0.5-1.5Hz, see Methods) for the one example where significant entrainment was observed (B), and another typical example (right). Time interval analyzed starts (at 0s) immediately after the end of trapezoidal TACS. No electrodes were found to be entrained in the frequency range after FDR correction.

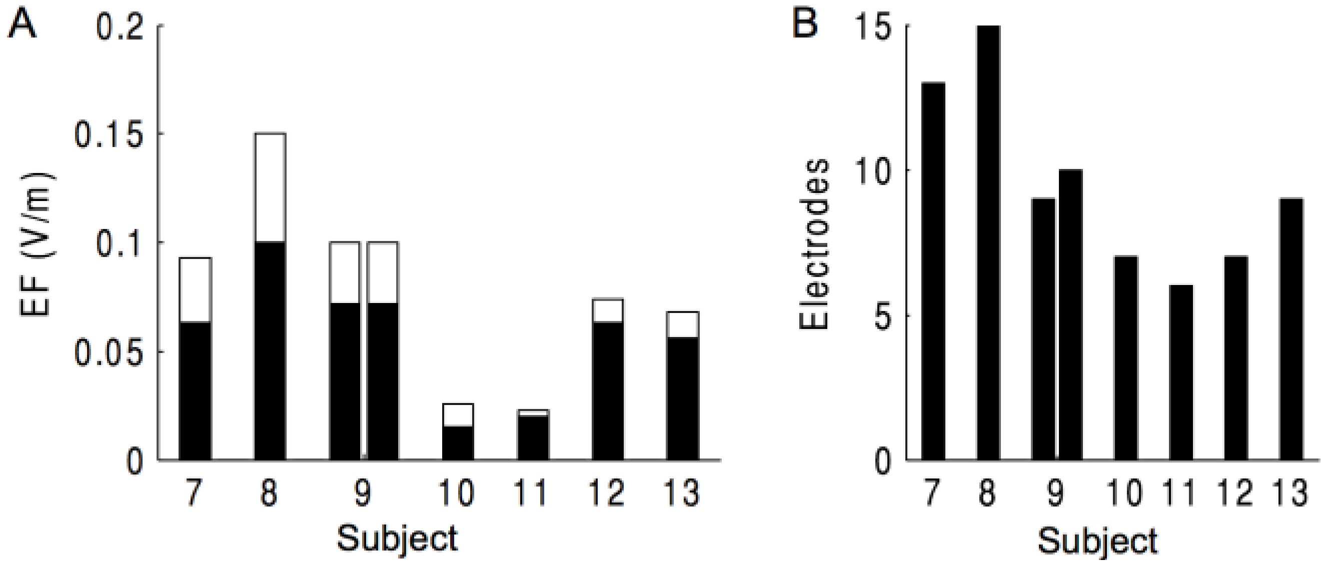

Supplementary Figure 14. Preselection of electrodes by magnitude of electric field. A) Maximum electric field magnitude (white bar) and 75th percentile (black bar) across electrodes. Electrodes in the top 25th percentile of electric field were selected. B) Number of electrodes with electric field magnitude in this top 25th percentile. These electrodes were analyzed and corrected for multiple comparisons using FDR. The two bars for subject S9 correspond to electrodes with usable data during stimulation at 1Hz and 0.75 Hz respectively.

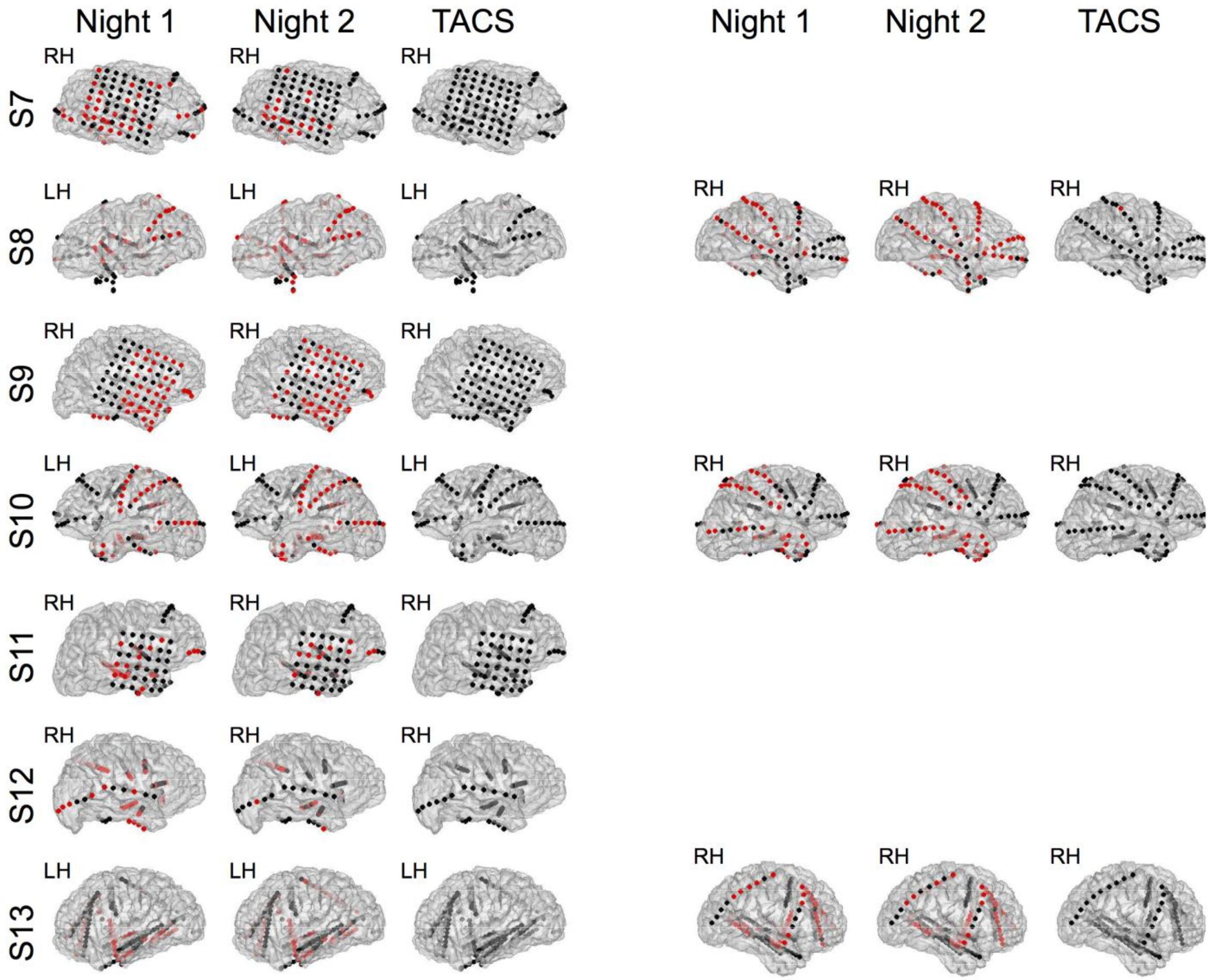

Supplementary Figures 15. Location of electrodes which demonstrated 14 Hz spindle entrainment during two nights of endogenous sleep and during TACS. Each row displays the location of the electrodes for each subject. Electrodes with significant spindle entrainment after multiple corrections are displayed in red. Subject S8, S10 and S13 have electrode coverage across both hemispheres, therefore the opposite view of the brain is shown on the right side. Multiple comparisons during TACS is performed only on the electrodes that are consistently entrained during both nights. During TACS, there are only two electrodes significant after multiple comparisons (subject S8). These electrodes are entrained during different stimulation blocks. The location of entrainment is not consistent, only the electrode entrained during the first stimulation block is displayed for subject S8.

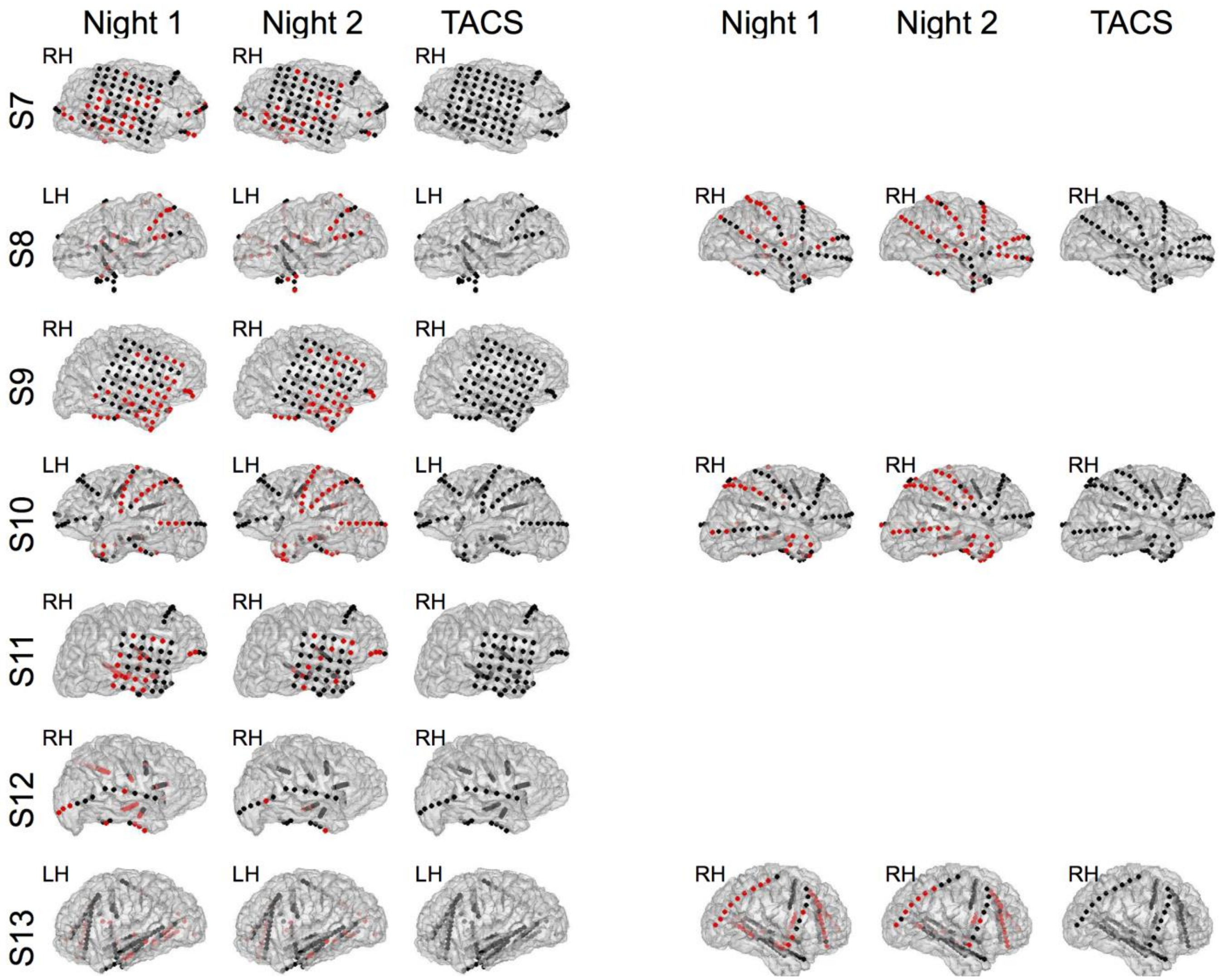

Supplementary Figure 16. Location of electrodes which demonstrated 10 Hz spindle entrainment during two nights of endogenous sleep and during TACS. Each row displays the location of the electrodes for each subject. Electrodes with significant spindle entrainment after multiple corrections are displayed in red. Subject S8, S10 and S13 have electrode coverage across both hemispheres, therefore the opposite view of the brain is shown on the right side. This figure demonstrates that multiple electrodes demonstrated slow spindle Multiple comparisons during TACS is performed only on the electrodes that are consistently entrained during both nights. During TACS, there is only one electrode significant after multiple comparisons (subject S13). These electrodes are entrained during different stimulation blocks. The location of entrainment is not consistent, only the electrode entrained during the third stimulation block is displayed for subject S13.

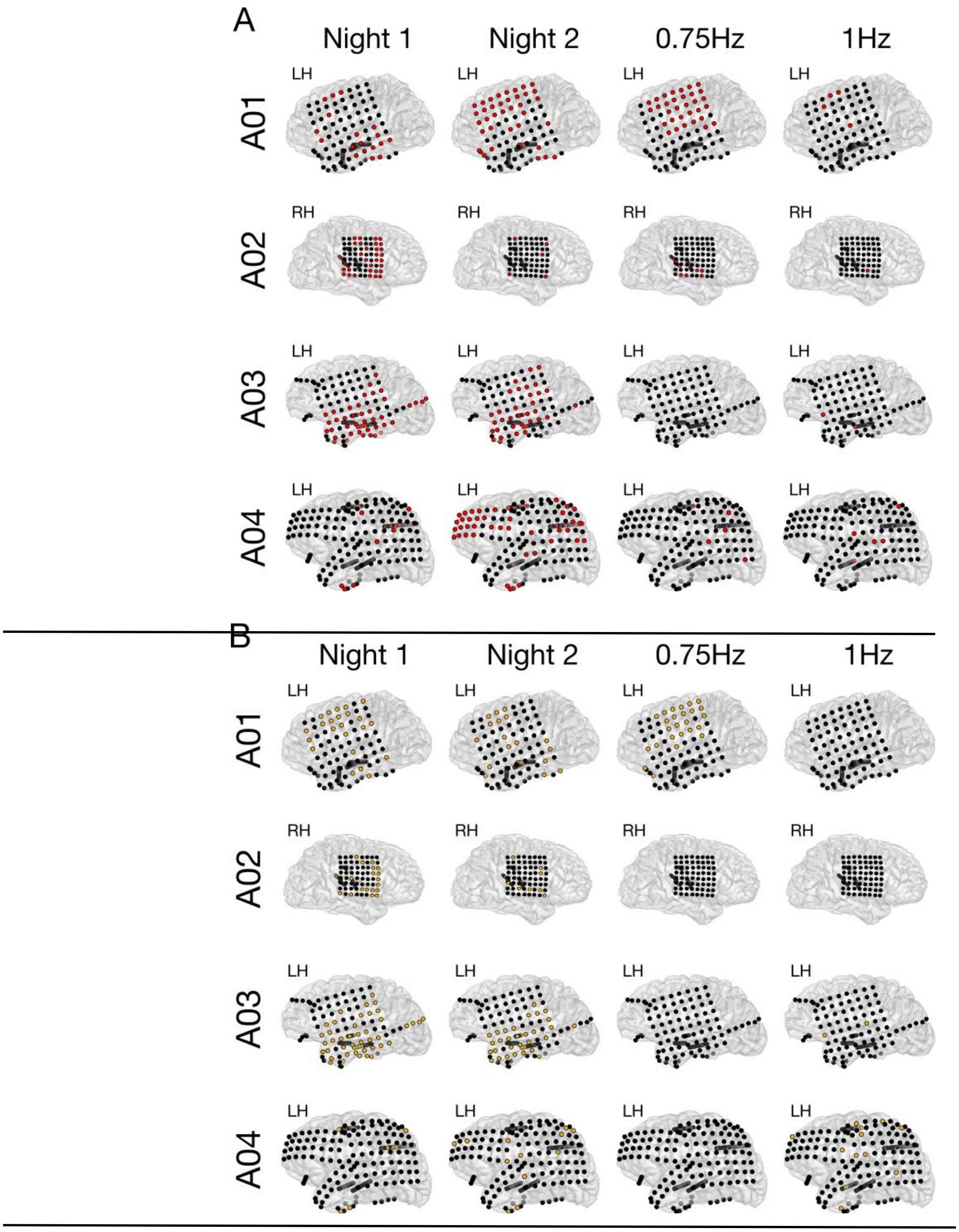
A
Night 1
Night 2
0.75Hz
1Hz
A01
A02
A03
A04
LH
RH
B
Night 1
Night 2
0.75Hz
1Hz
A01
A02
A03
A04
LH
RH

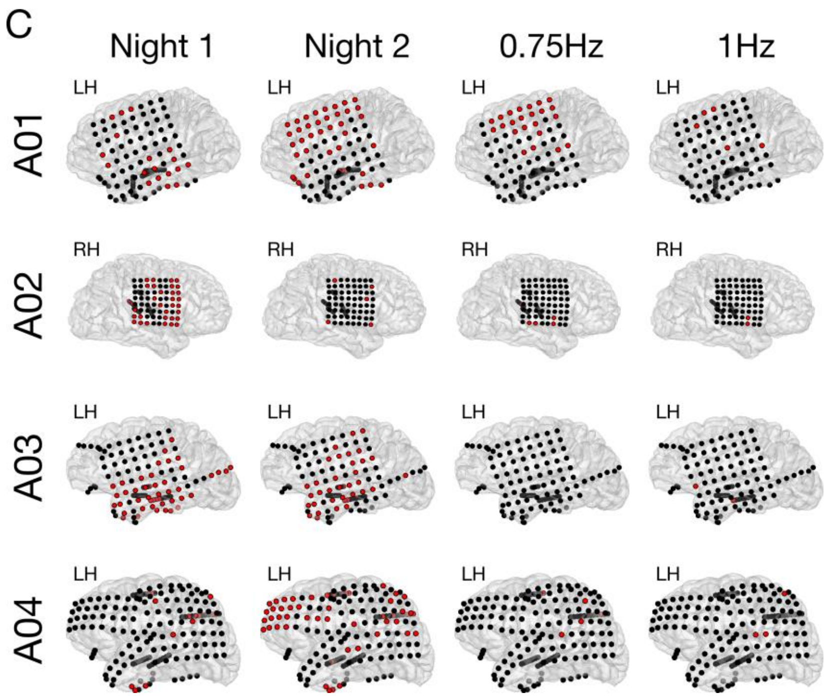

Supplementary Figure 17. Location of electrodes which demonstrated spindle (14 Hz, 10 Hz) entrainment during two nights of endogenous sleep and during 0.75Hz and 1Hz acoustic stimulation. Each panel shows the electrode locations for each subject (rows) **A:** Electrodes with significant 14Hz spindle entrainment after multiple corrections are displayed in red. **B:** Electrodes which showed significant 10Hz spindle entrainment after multiple comparisons are displayed in yellow. **C:** Same as top panel (14 Hz) except that multiple corrections for acoustic stimulation blocks were only applied to electrodes showing significant entrainment during Night 1 or Night 2.

SUPPLEMENTARY TABLES

Supplementary Table 1. Subject Characteristics. Subjects included were epilepsy patients with medication refractory seizures undergoing invasive monitoring at a single center. Demographic and clinical characteristics, electrode coverage, and stimulation characteristics are summarized. Electrocorticography grids have 64 electrode contacts, strips have between 4-10 electrode contacts, and depths have between 6-8 contacts.

Sub	Age	Sex	Hand-edness	Prior Surgery	Electrode Coverage	Stim	Freq	# of stim. blocks	Current	Meds	# electrodes tested
S1	27	M	--	N	LEFT:<br>8 strips (frontal, temporal, central, parietal, occipital)<br>2 depths (mesial temporal)<br>RIGHT:<br>9 strips (frontal, temporal, central, parietal, occipital, subgaleal)	Awake	1Hz	2 (10 min), 1 (5 min)	1, 1.25 mA	Lamotrigine<br>Clobazam<br>Eslicarbazepine	122

S2	29	F	R	N	LEFT:<br>1 grid (frontotemporal)<br>9 strips (frontal, temporal, parietal, occipital)<br>2 depths (mesial temporal)<br>RIGHT:<br>8 strips (frontal, temporal, parietal, occipital, subgaleal)	Awake	1Hz	2 (10 min)	0.75, 0.5 mA	Levetiracetam<br>Clobazam	112
S3	36	M	R	N	LEFT:<br>8 strips (frontal, temporal, parietal, occipital)<br>2 depths (mesial temporal)<br>RIGHT:<br>9 strips (frontal, temporal, parietal, occipital, subgaleal)<br>2 depths (mesial temporal)	Awake	1Hz	4 (10 min)	0.5, 1 mA	Felbatol<br>Eslicarbazepine<br>clobazam	117
S4	41	M	R	Right anterior temporal resection	RIGHT:<br>10 depths (frontal, temporal)	Awake	1Hz	4 (10 min)	1 mA	phenobarbital	78
S5	44	M	R	N	LEFT:<br>10 strips (frontal, temporal, parietal, occipital)	Awake	1Hz	4 (10 min)	0.75, 1 mA	Carbamazepine<br>klonopin	111
S6	36	F	R	N	LEFT:<br>1 grid (frontotemporal)<br>8 strips (frontal, temporal, parietal, occipital)<br>2 depths (mesial temporal)<br>RIGHT:<br>7 strips (frontal, temporal, parietal, occipital)	Awake	1Hz	3 (10 min)	0.75, 1 mA	Lacosamide	155
S7	30	F	R	Right anterior temporal resection	RIGHT:<br>1 grid (frontotemporal)<br>9 strips (frontal, temporal, parietal, occipital, subgaleal)<br>4 depths (mesial temporal, insula)	Sleep/ Wake	1Hz	6 (5 min)	1, 1.5 mA	Levetiracetam<br>Lacosamide	84
S8	42	F	R	Left anterior temporal resection	LEFT:<br>4 strips (frontal, temporal, parietal, subgaleal)<br>3 depths (frontal, insula)<br>RIGHT:<br>9 strips (frontal, temporal, central, parietal, subgaleal)<br>2 depths (mesial temporal)	Sleep/ Wake	1Hz	6 (5 min)	1.5, 2 mA	Eslicarbazepine<br>Lamotrigine	116

S9	57	M	R	N	RIGHT:<br>1 grid (centrotemporal)<br>4 strips (frontal, temporal)<br>2 depths (mesial temporal)	Sleep/<br>Wake	1Hz,<br>0.75 Hz	6 (5 min)	1.5 mA	Off meds	89
S10	22	M	R	N	LEFT:<br>8 Strips (2 frontal, 2 temporal, 2 parietal, 1 occipital, 1 subgaleal)<br>5 Depths (sub-genu, mesial frontal, mesial temporal, mesial parietal)<br>RIGHT:<br>8 Strips (2 frontal, 2 temporal, 2 parietal, 1 occipital, 1 subgaleal)<br>5 Depths (sub-genu, mesial frontal, mesial temporal, mesial parietal)	Sleep/<br>Wake	1Hz	2 (5 min)	0.3, 1, 1.5, 2.0 2.5 (woke up with stimulation at 2.5)	Off meds	93
S11	22	F	R	N	RIGHT:<br>1 grid (temporal)<br>5 depths (temporal)<br>4 strips (anterior frontal, inferior frontal, anterior temporal, mesial temporal)	Sleep	0.75 Hz<br>TES	4 (5 min)	0.3 mA (felt stimulation at 0.4 mA)	Levetiracetam<br>Lacosamide	83
S12	24	M	R	Right temporal resection	RIGHT:<br>4 strips (3 temporal, 1 occipital)<br>7 depths (2 mesial temporal, 3 frontal, 1 central, 1 parietal)	Sleep	1 Hz TES	4 (5 min)	0.5/1.75 mA	Lamotrigine	49
S13	25	M	R	N	LEFT:<br>9 depths (2 frontal, 2 insular, 5 temporal)<br>RIGHT:<br>10 depths (5 frontal, 1 insular, 4 temporal)	Sleep	0.75 Hz,<br>1 Hz TES	8 (5 min)	1.5, 2 mA	Phenobarbital	79
S14	32	M	R	N	LEFT<br>8 strips (4 frontal, 1 temporal, 2 parietal, 1 occipital)<br>2 depths (insular)<br><br>RIGHT<br>8 strips (4 frontal, 1 temporal, 2 parietal, 1 occipital)<br>2 depths (insular)	Sleep	0.75 Hz<br>Trapezoidal 0.26 mA<br>F3/F4<br>8 mm ring electrodes		0.26 mA	Clobazam	113
A01	17	M	R	N	LEFT:<br>1 grid (frontotemporal)<br>5 strips (1 frontal, 3 temporal)<br>3 depths (mesial temporal)	Sleep	0.75, 1 Hz<br>Acoustic	1,1 (5-min)	N/A	Lamotrigine<br>Eslicarbazepine	110
A02	26	M	R	N	RIGHT:	Sleep	0.75, 1	1,1 (5min)	N/A	Topiramate,	94

					1 grid (frontoparietal)<br>4 depths (parietal)		Hz<br>Acoustic			Lamotrigine<br>Oxcarbazepine	
A03	58	F	R	N	LEFT:<br>1 grid (frontoparietal)<br>6 strips (2 frontal,3 temporal, 1 occipital)<br>3 depths (2 mesial temporal,1 insular)	Sleep	0.75, 1 Hz<br>Acoustic	1,1 (5min)	N/A	Lamotrigine, Zonisamide	118
A04	22	M	R	N	LEFT:<br>2 grids (frontotemporal, parieto-occipital)<br>4 strips (1 frontal, 3 temporal)<br>4 depths (mesial temporal, supplementary motor, precuneus)	Sleep	0.75, 1 Hz<br>Acoustic	1,1 (5min)	N/A	Off meds	126

Supplementary Table 2. Entrainment of spindle activity by endogenous slow oscillations during two different nights. Results are shown for cross frequency coupling analysis (14 Hz, columns 4-6; 10 Hz, columns 8-10) for 11 subjects (S7-S13; A1-A4).

Subject	Night 1	Night 2	PAC N1 (14 Hz)	PAC N2 (14 Hz)	# Electr. consistent N1&N2 PAC, 14Hz	Degree (14Hz)	PAC N1 (10 Hz)	PAC N2 (10 Hz)	# Electr. consistent N1&N2 PAC, 10Hz	degree (10 Hz)
S7	23 min	29 min	43% (53/123)	22% (27/121)	25	1±2	33% (41/123)	26% (32/123)	23	-10±3
S8	26 min	28 min	54% (56/103)	75% (85/113)	52	8±4	39% (40/103)	48% (54/113)	27	23±5
S9	45 min	45 min	61% (59/96)	71% (64/89)	52	-3±2	50% (48/96)	56% (50/89)	36	-10±3
S10	14 min	30 min	69% (63/91)	94% (90/96)	65	-3±2	52% (47/91)	79% (76/96)	41	-6±2
S11	30 min	30 min	54% (38/70)	43% (35/82)	14	1±9	61% (43/70)	34% (28/82)	15	-6±23
S12	60 min	30 min	81% (76/94)	35% (33/93)	32	-70±8	62% (58/94)	3% (3/93)	3	-49±5
S13	30 min	30 min	93% (159/170)	81% (146/164)	145	-1±2	79% (135/170)	41% (68/164)	64	-3±2

A1	30 min	30 min	50% (55/110)	78% (86/110)	44	44±0	51% (56/110)	58% (64/110)	41	-2±11
A2	30 min	30 min	53% (50/94)	9% (8/94)	6	-35±1	27% (25/94)	15% (14/94)	9	-2±7
A3	30 min	30 min	59% (70/118)	47% (55/118)	41	-0.6±0.4	64% (75/118)	43% (51/118)	41	-2±6
A4	30 min	30 min	14% (18/126)	50% (63/126)	14	32±1	13% (17/126)	13% (16/126)	4	-2±6

SUPPLEMENTARY NOTES

Supplementary Note 1. Slow wave activity is broad band with short coherence time

The autocorrelation of the raw signal was analyzed for all subjects during sleep to determine if there is a clear slow wave oscillation with multiple oscillation cycles. In Supplementary Figure 5, the analysis is shown for subject S7. Only a few electrodes show a second peak in the autocorrelation at approximately 1 sec of positive and negative time lag, indicating that slow wave oscillations are steady only for about two cycles in those electrodes. For most electrodes, the slow wave activity is a unitary event (not oscillatory). Indeed, the power spectrum during sleep indicates that the peak of slow wave activity is broadband as a result of this short coherence time as shown in Supplementary Figure 6.

Supplementary Note 2. Sleep spindles are coupled to phase of endogenous slow oscillations also at 0.75 Hz

Sleep iEEG data from the seven subjects (S7-S13) were analyzed over the two nights. We repeated the analysis described in the result section, testing for modulation of spindle amplitude (fc=14 Hz, bandwidth=7 Hz) with the phase of by the endogenous slow oscillations (fc=0.75 Hz, bandwidth=0.75 Hz) in all subjects. Of the 472 electrodes tested across subjects, 329 exhibited significant PAC (FRD, q<0.05). Supplementary Figure 7 show the preferred phase of fast spindle relative to the 0.75Hz phase in two nights of (non-stimulated) NREM sleep. 76% of the channels showing PAC during night 1 also demonstrated PAC during night 2.

Supplementary Note 3. Considerations on statistical power

No preliminary data for effect size was available for iEEG to allow for a formal power analysis. For human scalp EEG, the question of entrainment has been addressed in Reato, 2013[1], were we reanalyzed data for the original Marshall et al study[2]. There 13 subjects with 5 repetitions each of 10 seconds of endogenous activity following TACS were used to assess entrainment. As we show in Supplementary Figure 5 coherence is preserved for at most 2 cycles. This means that at most 2 cycles

per trial after stimulation remain with a consistent phase to meaningfully contribute to the statistical test (the same test we used here also for the replication study on patient S14; see Supplementary Figure 13). This gives a total of 160 cycles that contribute meaningfully to the statistical power of the entrainment test. In contrast, here, we tested in block of 5 minutes giving 300 cycles. Thus, we expected with the better data quality to find effects in each block tested. In contrast to Reato, 2013[1] we had many more electrodes (over 100 instead of 11) and thus correction for multiple comparisons possibly reduced the power of the test. A point of reference is the analysis of endogenous slow-wave oscillations, where despite multiple comparison correction we find significant entrainment of spindle and gamma activity with the slow-wave oscillation for a large fraction of electrodes (between 17% to 77% of electrodes tested). There we used approximately 20 minutes of NREM sleep for analysis. This is comparable to the durations we used when combining all TACS blocks, which gave a combined 10-30 minutes for analysis in each subject. Given that we had intracranial recording we expected effects to be significantly more robust. Indeed, when using only 5-minute segments of endogenous slow wave oscillations we found entrainment in approximately 10% of electrodes, which attests to the statistical power of the test used.

Supplementary Note 4. Ruling out alternative explanations

To exclude the possibility that we missed a genuine effect, we performed a number of additional post-hoc analyses with less conservative thresholds, and using different sub-selection criteria as follows:

Applying a less conservative statistical threshold. All tests for significant PAC were corrected for multiple comparisons across electrodes using FDR correction. To exclude the possibility that our threshold for significance was too stringent, we looked at uncorrected p-values for tACS, endogenous sleep, and acoustic stimulation when analyzing fast spindle (14Hz) amplitude (Supplementary Figures 8-10). During electrical stimulation, only a 21.5% of electrodes tested have uncorrected $p<0.05$. Importantly, only 2.5% show uncorrected $p<0.05$ in more than one stimulation segments (Supplementary Figure 8). In contrast, for endogenous slow-waves (no-stimulation) 61.2% are at uncorrected $p<0.05$, with 47.4% of the electrodes consistently entrained at uncorrected $p<0.05$ during both nights (Supplementary Figure 9). Similarly, a larger fraction of electrodes, 20% are entrained to acoustic stimulation with $p<0.05$, with 7% consistent across stimulation blocks with 1Hz and 0.75 Hz noise-burst rate (Supplementary Figure 10).

Absence of spindle entrainment in the electrodes with the highest measured electric fields. It is possible that a subset of electrodes entrain in their spindle amplitude to the slow oscillations, but that this effect is lost due to correction for multiple comparisons. To account for this possibility, in an additional post-hoc analysis we constrain FDR correction only to those electrodes with the highest measured field strengths (top 25th percentile, Supplementary Figure 14). We reasoned that an effect is likely to be

present only where fields are sufficiently strong. These electrodes were primarily in the frontal cortex. No significant PAC between fast spindle amplitude and tACS phase was found in any subject (at p<0.05), except for subject S8, after FDR correction. In S8, 1 out of the 15 electrodes (sub selection of electrodes that experience an electric field magnitude in the 25th percentile) showed significant PAC (N=292 cycles, p=0.0003 uncorrected), however the effect was not stable, as it was only observed on 1 out of 6 stimulation blocks.

Absence of tACS-induced spindle entrainment in the electrodes demonstrating spindle entrainment during endogenous sleep. It is possible that entrainment of slow wave activity via tACS is highest and/or limited to those electrodes which naturally show strongest modulation of spindle amplitude. To address this alternative, we limited the analysis to the N=369 electrodes which demonstrated stable entrainment of spindle oscillations during endogenous NREM sleep (Supplementary Figure 15 and 16 for 14 Hz and 10 Hz respectively). This sub-selection, however, does not increase the number of electrodes that cross significance after multiple comparison corrections for at least one block (2 of 445 electrodes tested with sub-selection vs 2 of 769 electrodes without). Importantly, none of the electrodes where consistently entrained across blocks (with FRD, q<0.05 for the sub-selection). In contrast, for the case of acoustic stimulation there are still approximately 10-14% of electrodes that show significant entrainment (Supplementary Figure 17). This supports the interpretation that entrainment of spindle activity to periodic acoustic stimulation is related to entrainment of the endogenous slow-wave rhythm, and not simply the result of stimulus induced slow-wave events.

Absence of power changes after a 5-minute block of stimulation compared to before stimulation. Previous studies have reported increased spindle power after a 5-minute block of tACS[2, 3, 4, 5, 6]. To investigate whether similar aftereffects could be observed intracranially, we compared slow wave and spindle power in the 30 seconds immediately preceding and following each 5-minute stimulation block. We focused on this period to account for the large variability of slow wave across the night and between nights. Statistical significance of changes in power were assessed using the Chronux toolbox (see Methods section "Analysis of changes in power before and after stimulation"). The results are mixed across electrodes and across subjects (Supplementary Figure 12). Most of the electrodes (76$\pm$19% mean across S7-S13) do not have a statistically significant difference in power (show in green). Electrodes with significant differences in power (FDR, q<0.05) have mixed behaviors: 13$\pm$10% electrodes across S7-S13 show an increase and 11$\pm$10% a decrease (displayed in red/blue for increase/decrease respectively). Some subjects showing an increase (S11, S13/0.75 Hz), some decrease (S8), some have mixed results across electrodes (S7, S9/0.75 Hz, S13/1Hz) or with only few electrodes showing changes (S9/1Hz, S12). There was no systematic trend that could be ascribed to the tACS. Instead, the fluctuations observed are consistent with natural fluctuations expected during sleep on the timescale of 15-40 minutes used for stimulation[1].

Supplementary References

1. Reato D, Gasca F, Datta A, Bikson M, Marshall L, Parra LC. Transcranial electrical stimulation accelerates human sleep homeostasis. *PLoS Comput Biol* **9**, e1002898 (2013).

2. Marshall L, Helgadóttir H, Mölle M, Born J. Boosting slow oscillations during sleep potentiates memory. *Nature* **444**, 610-613 (2006).

3. Westerberg CE*, et al.* Memory improvement via slow-oscillatory stimulation during sleep in older adults. *Neurobiol Aging* **36**, 2577-2586 (2015).

4. Prehn-Kristensen A*, et al.* Transcranial oscillatory direct current stimulation during sleep improves declarative memory consolidation in children with attention-deficit/hyperactivity disorder to a level comparable to healthy controls. *Brain Stimulation* **7**, 793-799 (2014).

5. Goder R, Born J. Can sleep heal memory? *Sleep Med Rev* **17**, 89-90 (2013).

6. Ladenbauer J*, et al.* Promoting Sleep Oscillations and Their Functional Coupling by Transcranial Stimulation Enhances Memory Consolidation in Mild Cognitive Impairment. *J Neurosci* **37**, 7111-7124 (2017).
